# Theory of Mind Development in School-Aged Left-Behind Children in Rural China

**DOI:** 10.3389/fpsyg.2018.01819

**Published:** 2018-09-21

**Authors:** Yanchun Liu, Xuelian Yang, Jingjing Li, Erhu Kou, Huidong Tian, Heqing Huang

**Affiliations:** ^1^College of Education Science, Hubei Normal University, Huangshi, China; ^2^Wuhan Sports University, Wuhan, China; ^3^College of Preschool Education, Capital Normal University, Beijing, China

**Keywords:** theory of mind, left-behind, school-aged children, rural areas, protective factor

## Abstract

The current study aimed to investigate differences in theory of mind between left-behind children and non-left-behind children in rural China and to examine the potential protective role of general reasoning ability in left-behind children’s theory of mind. Participants included 213 children aged 7.10–13.67 years (111 boys and 102 girls, *M* = 10.51 years, *SD* = 1.33), 101 of whom were left behind in rural areas by one or both migrating parents for at least 6 months. The Strange Stories task, a second-order false belief task, and a faux pas task were used to measure children’s theory of mind, and Sessions B and C in Raven’s Standard Progressive Matrices were used to test children’s general reasoning ability. The results showed that left-behind children scored lower on both the faux pas task and Strange Stories task. Additionally, on second-order false belief understanding, left-behind boys performed worse than non-left-behind boys, while left-behind girls scored higher than non-left behind girls. Moreover, children’s general reasoning ability moderated the relationship between parental migrant status and children’s faux pas understanding: For children with high levels of general reasoning ability, left-behind children performed similarly to non-left-behind children, while for children with low levels of general reasoning ability, left-behind children scored lower than non-left-behind children, indicating that general reasoning ability buffered the negative effect of being left behind on children’s theory of mind development. The implications of these findings for training directed at left-behind children are discussed.

## Introduction

Since 1978, China’s economy has rapidly progressed due to the reform and open-door policy (Jaggi et al., 1996), which facilitated the process of urbanization. As a result, massive numbers of rural surplus laborers have migrated from rural areas to large cities for better jobs and higher salaries, which has been described as the largest migration in human history (Zhang, 2004). Unfortunately, the combined forces of parents’ long working hours in urban areas, indigent living conditions, and insufficient income have led to unprecedented growth in the number of children left in their rural hometown by their migrating parents (Luo et al., 2009). In China, children under 18 years old are defined as left-behind children if they “have been left behind at their original residence while one or both parents migrate into other places for work, and have been not living together with them for at least six months" (Zhou and Duan, 2006). Non-left-behind rural children are defined as “children under 18 who live with both parents at their original residence." According to China’s Sixth National Population Census, China has nearly 69.7 million left-behind children, 61 million of whom live in rural areas. This means that left-behind children from rural areas comprise 87.5% of Chinese left-behind children and approximately 22% of China’s total child population (Duan et al., 2013; Ding and Bao, 2014). Compared with non-left-behind children, left-behind children are confronted with less parental control and supervision, less parental support and guidance, and weaker parent–child bonding (Wen and Lin, 2012). These disadvantages negatively influence left-behind children’s emotional development (Jia and Tian, 2010; He et al., 2012), self-awareness (Wang et al., 2014; Sun et al., 2015), mental health (Zhao and Yu, 2016), social behaviors (Hu et al., 2014), and so on. Clearly, left-behind children represent a new vulnerable group that should not be ignored. To date, however, few studies have focused on left-behind children’s theory of mind development (Zhao and Shi, 2009). Thus, adopting a comparative approach, this study aims to contribute to the literature by presenting the empirical patterns of the theory of mind development of school-aged left-behind children, in comparison with those of non-left-behind children, in rural communities in China.

Theory of mind refers to reasoning about mental states such as intentions, desires, beliefs, feelings, and emotions, and it also refers to explaining behaviors in accordance with individual mental states (Premack and Woodruff, 1978), which not only plays a vital role in social interactions (Keenan, 2003; Paal and Bereczkei, 2007; Hao and Liu, 2016) but also has an influence on children’s school achievement (Garner and Waajid, 2008; Lecce et al., 2017). Previous research on theory of mind developmental trajectories has indicated that by age 4–5 years, children can pass first-order false belief tasks – that is, they can differentiate people’s internal beliefs from reality (Wellman et al., 2001). A growing body of literature indicates that the theory of mind continues to become increasingly sophisticated starting in early middle childhood (Miller, 2012). Second-order false belief tasks, in which children are required to infer about one agent’s false belief regarding another agent’s true or false belief, are broadly used to examine school-aged children’s theory of mind development (Perner and Wimmer, 1985). Most children can pass these tasks when they are 9 or 10 years old (Perner and Wimmer, 1985). Additionally, researchers have used the Strange Stories task to test multiple types of theory of mind understanding among children, such as lie, white lie, double bluff, irony, and persuasion understanding, and found that 8-year-old children can understand these mental states (Happé, 1994; Bussey, 1999; Filippova and Astington, 2008). Moreover, children become more sophisticated in identifying whether and why a person makes a faux pas in conversation from age 9 to age 11 years (Baron-Cohen et al., 1999; Banerjee et al., 2011). Thus, this study included all of the abovementioned tasks to paint a full picture of Chinese rural left-behind children’s theory of mind development, in comparison with that of their non-left-behind counterparts.

According to social cognitive theory (Carpendale and Lewis, 2004), social experience is one of the most important origins of individual differences in children’s theory of mind development. Interactions with others allow children to reflect on their social experiences and increase their realizations that people can have different perspectives on the same situation (Carpendale and Lewis, 2004; Nelson, 2007; Lecce et al., 2014; Bianco et al., 2016). Considerable empirical evidence suggests that social experience with their parents contributes to preschoolers’ theory of mind development (Ruffman et al., 2002; Symons, 2004; Hughes et al., 2005; Pavarini et al., 2013; Liu et al., 2016), and some studies have found that parent–child communication is important for school-aged children’s theory of mind development (Ensor et al., 2014). Studies about school-aged deaf children also provide support for the role of parent–child communication in children’s theory of mind. School-aged deaf children born to hearing parents exhibited delayed theory of mind success, but those born to deaf parents did not (Peterson and Siegal, 1995; Peterson and Slaughter, 2006; Hao and Su, 2014) because the former lacked social experiences with their parents. Nevertheless, compared with their non-left-behind counterparts, left-behind children have limited opportunities for communicating with their parents (Li, 2002; Zhao and Yu, 2016). This limited participation in social interactions with parents may impose restrictions on children’s opportunities to learn about mental states. Thus, we can infer that left-behind children would perform worse than non-left-behind children on theory of mind tasks. To date, it is unclear whether and how the left-behind experience has influenced children’s theory of mind, which is the first purpose of this study.

Resilience research indicates that risk factors do not always have negative effects on children’s development, and children under adversity have opportunities to develop normally or even outperform other children if there are protective factors for them (Luthar et al., 2000; Werner and Smith, 2001). Lerner et al. (2009) proposed that risk factors have negative effects on the developmental outcomes of only approximately 20–49% of high-risk children, while protective factors appear to predict positive developmental outcomes for approximately 50–80% of such children. Compared to those without a particular protective attribute, individuals with the attribute were relatively unaffected by adversity. Luthar et al. (2000) proposed three types of protective factors, including community (e.g., neighborhood and social support), family and peers, and the child’s characteristics (gender, reasoning ability, or social skillfulness). In this study, gender was considered a protective factor for children’s theory of mind. Several studies have already provided evidence of girls’ superiority in theory of mind (Charman et al., 2002; Walker, 2005; Calero et al., 2013; Devine and Huges, 2013). The sex-segregated peer play is typical in middle childhood that girls are more likely to develop close friendships with a small number of female peers while boys tend to socialize in larger male groups (Maccoby, 1966). Conversational turn taking and expressing the agreement and approval of others’ perspectives are more demanding in interactions within female groups, which may make it possible for girls to practice their theory of mind (Maccoby, 1990). Left-behind girls have more opportunities and time to develop relationships with their peers due to the limited parental supervision (Sun et al., 2010), which provides more opportunities for them to refine their theory of mind. Thus, left-behind girls may outperform in theory of mind in comparison to their counterparts.

According to resilience theory (Luthar et al., 2000; Werner and Smith, 2001), children’s general reasoning ability may be another protective factor for children’s theory of mind development. First, many studies have documented the important role of general reasoning ability in children’s theory of mind development (Wilhelm et al., 2010; Ibanez et al., 2013; Baker et al., 2014). Second, left-behind children would get more care and conversations from their teachers, which is especially true for those with high levels of general reasoning abilities (Liu et al., 2015). Finally, cognitively developmental children would better accept their parents’ migration (Sison and Arellano-Carandang, 2007), which may enable them to comprehensively consider their parents’ perspectives and their own views. Thus, general reasoning ability may serve as one of the protective factors in left-behind children’s theory of mind development. Examining the potentially protective role of gender and general reasoning ability in left-behind children’s theory of mind development is the second purpose of this study.

In summary, the current study compared left-behind children with children from non-migrant families in rural communities of Hubei Province in China to understand the similarities and differences in their theory of mind. The potential protective effects of general reasoning ability on left-behind children’s theory of mind were also examined. We hypothesized that (1) due to limited opportunities to interact with their parents, left-behind children would perform poorer on theory of mind tasks; (2) considering the characteristics of sex-segregated peer play in middle childhood and the more opportunities and time for left-behind girls to engage in peer interactions, left-behind girls would perform better than their counterparts on theory of mind tasks; and (3) based on the resilience theory, children’s general reasoning ability would play a predictive role in theory of mind development: for children with high levels of general reasoning ability, left-behind children would perform similarly to non-left-behind children on theory of mind tasks, while for children with low levels of general reasoning ability, left-behind children would score lower than non-left behind children. To our knowledge, the current study is the first to examine mental understanding by comparing rural left-behind children and their counterparts from non-migrant families in China.

## Materials and Methods

### Participants

The participants were from rural counties of Hubei Province, the site of a large laborer migration in China. A total of 213 children (111 boys and 102 girls) aged 7.10–13.67 years participated in this study, *M*_age_ = 10.51 years, *SD* = 1.33. They included 101 left-behind children (one or both parents had migrated for work for at least 6 months; 58 boys and 43 girls) and 112 children (53 boys and 59 girls) who lived with both parents. The left-behind and non-left-behind children groups were balanced in gender, χ^2^(1) = 2.17, *p* = 0.170. Among the left-behind group, 48 children were from both-parent-migrant families and lived with their grandparents, and 53 were from single-parent-migrant families and lived with the other parent who did not migrate. All children were of Han nationality. All procedures performed in the studies involving human participants were in accordance with the ethical standards of the institutional and/or national research committee and with the 1964 Helsinki declaration and its later amendments or comparable ethical standards. All procedures were approved by the Research Ethics Board of the Department of Psychology in Hubei Normal University. Informed written consent was obtained from the children and their parents or other guardians included in the study.

### Procedures

Children were assessed by trained research assistants individually in a quiet room. Happé (1994) Strange Stories Test, adapted by Hao and Liu (2016), a second-order false belief task (Happé et al., 1998), and a faux pas task (Wang and Su, 2013) were used to examine different types of theory of mind. Sessions B and C in Raven’s Standard Progressive Matrices (RSPM) were used to test children’s reasoning ability. The order of each theory of mind task and RSPM was randomized. Each child’s basic information (e.g., who migrated for work, how long had he/she/they been away from home) was collected from their parents or guardians. All the tasks were completed in approximately 30–40 min.

Strange stories. Five stories were used to test children’s understandings of lie, white lie, double bluff, irony, and persuasion (Happé, 1994; Hao and Liu, 2016). Following Hao and Liu (2016) procedure, the research assistants told the stories loudly, and a cartoon-style illustration accompanied the stories to eliminate children’s memory load. After each story, children were asked to answer why the protagonist said what he/she had said. In line with the scoring standard in Happé et al. (1998), full and correct answers that explicitly referred to mental states were scored 2. Partial answers that implicitly mentioned the correct mental states were scored 1. Incorrect and unrelated answers were scored 0. Thus, the range of scores for each story was 0–2, and the total score ranged from 0 to 10.

Second-order false belief task. We modified the stories by Happé et al. (1998) to use a context and character names that were familiar to Chinese children. Children were told the following story (with illustrations): Ming and Hong saw an old man selling ice cream in a park. Ming was eager to taste the ice cream, but neither of them had any money, so Ming went home to get money. Unfortunately, the seller left the park and went to the school nearby to sell ice cream, and he said to Hong that he would meet him at the school. Hong went to Ming’s house to tell him where the old man went, but he found Ming had left the home. Then, the children were asked two test questions: “Where would Ming go to meet with Hong?” and “Why would Ming go there?” Correct answers received 1 point, so the scores on this task ranged from 0 to 2.

Faux pas task. The “broken vase” story from Wang and Su (2013) was used to examine children’s faux pas understanding. In this story, the “insulting” character, Qian, unintentionally insulted a crystal vase that the “insulted” character, Ling, had sent to Qian as a present. Qian was ignorant to Ling’s relation with the vase. Four test questions listed below were presented in a fixed order:

1. Did anyone say something they shouldn’t have said in the story?
2. Who said something they should not have said in the story?
3. Why shouldn’t the individual in the story say what they did?
4. Why do you think they did say it?

Questions 1 and 2 referred to the faux pas identification, and the correct answer was “yes” for Question 1 and “Qian” for Question 2. Question 3 involved appreciation for the listener’s emotional state or feelings, and Question 4 involved the understanding of the “insulting” character’s intention. The scoring standards of Questions 3 and 4 were similar to those of the Strange Stories described above (Wang and Su, 2013). The total score on the faux pas tasks ranged from 0 to 6.

The first author of this study scored all the answers, and another rater blind to the purpose of this study scored 25% of all answers. The inter-rater reliability (kappa coefficients) for the lie, white lie, double bluff, irony, persuasion, second-order false belief, and faux pas stories were 0.95, 0.91, 0.91, 0.84, 0.85, 0.95, and 0.90, respectively. According to Nunnaly (1978) and De Vellis (1991), these values of agreement were high.

Raven’s Standard Progressive Matrices. The psychometric properties of the RSPM items have been thoroughly analyzed and are used as an indicator of general reasoning ability throughout the world (Raven, 1989). Session B and Session C in RSPM were used to test children’s general reasoning abilities. Each session included 12 items; thus, the total score for reasoning ability ranged from 0 to 24.

### Data Analysis

Multiple independent *t*-tests were conducted to preliminarily examine the group differences in the children’s ages, general reasoning ability, and performance in each theory of mind task. Then, several ANCOVAs were used to examine the interactions between the group and gender for the children’s scores in each type of theory of mind task, controlling for the children’s ages and general reasoning ability. Moreover, hierarchical regressions on each theory of mind task score were conducted to examine the potential protective effect of general reasoning ability. Finally, online resources^1^ were used to plot the interactive effects and simultaneously conduct simple slope tests.

## Results

Multiple independent sample *t*-tests showed that left-behind children from both-parent-migrant families performed similarly to those from single-parent-migrant families on theory of mind tasks and a general reasoning ability task. Thus, in the next analysis, we incorporated these two subgroups of children into the left-behind children group. **Table 1** shows the *mean and SD* of all types of theory of mind tasks by group. Several independent sample *t*-tests were conducted to preliminarily examine group differences in children’s theory of mind. The results showed that left-behind children scored lower than non-left-behind children on the Strange Stories task, *t*(211) = 2.45, *p* = 0.015, *d* = 0.34, on the faux pas tasks, *t*(211) = 2.33, *p* = 0.021, *d* = 0.32, and in reasoning ability, *t*(211) = 2.54, *p* = 0.012, *d* = 0.35. Based on these crude statistics, without controlling for any potential confounding factors, it appeared that left-behind children differed from non-left-behind children on most of the theory of mind tasks.

**Table 1:** Mean differences between left-behind children and non-left-behind children in theory of mind.

	LBC		NLBC		*t*	Cohen’s *d*
	*M*	*SD*	*M*	*SD*		
Age	10.56	1.40	10.47	1.28	0.52	0.07
Reasoning	12.95	5.89	15.00	5.90	2.54^∗^	0.35
Scores on strange stories	4.47	1.71	5.03	1.63	2.45^∗^	0.34
Second-order false belief	0.51	0.82	0.57	0.87	0.57	0.07
Faux pas understanding	3.08	1.42	3.56	1.59	2.33^∗^	0.32

*p < .05. LBC, left-behind children; NLBC, non-left-behind children.

**Table 2** shows the *mean and SD* for the key variables by group and gender. A series of 2 (gender: boy or girl) × 2 (group: left-behind or non-left-behind) ANCOVAs were conducted for children’s scores in each type of theory of mind task, controlling for the children’s ages and general reasoning abilities. For the scores on the Strange Stories task, the main effect of gender, *F*(1,207) = 0.60, *p* = 0.442, $\eta_{p}^{2}$ = 0.003, and the gender × group interaction, *F*(1,207) = 0.67, *p* = 0.413, $\eta_{p}^{2}$ = 0.003 were not significant, but the main effect of group was significant, *F*(1,207) = 4.30, *p* = 0.039, $\eta_{p}^{2}$ = 0.020.

**Table 2:** Means (standard deviations) of children’s performance on theory of mind tasks by group and gender.

	Boys		Girls	
	LBC	NLBC	LBC	NLBC
	(*n* = 58)	(*n* = 53)	(*n* = 43)	(*n* = 59)
Strange stories task scores	4.34 (1.87)	5.06 (1.55)	4.63 (1.48)	5.00 (1.71)
Second-order false belief	0.36 (0.72)	0.79 (0.95)	0.70 (0.91)	0.37 (0.74)
Faux pas understanding	3.01 (1.43)	3.68 (1.53)	3.16 (1.41)	3.46 (1.65)

*LBC, left-behind children; NLBC, non-left-behind children.*

**FIGURE 1:**
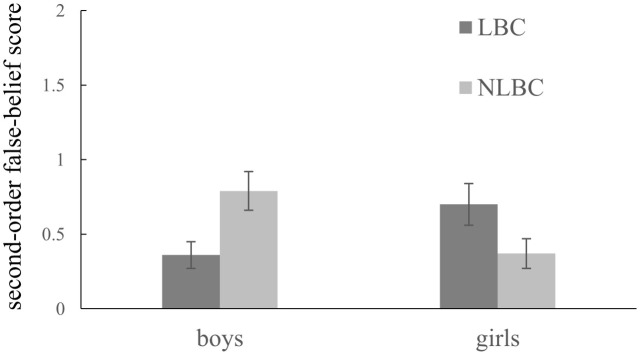
Second-order false belief scores as a function of gender and group (*N* = 213). The error bars added to each column represent standard errors in the figure. LBC represents left-behind children and NLBC represents non-left-behind children.

For second-order false belief scores, the main effects of gender, *F*(1,207) = 0.02, *p* = 0.877, $\eta_{p}^{2}$ < 0.001, and group, *F*(1,207) = 0.04, *p* = 0.846, $\eta_{p}^{2}$ < 0.001, were not significant, but the gender × group interaction was significant, *F*(1,207) = 11.47, *p* = 0.001, $\eta_{p}^{2}$ = 0.053. To further understand the interaction, we conducted two ANCOVAs to examine the gender differences within the groups of left-behind children and non-left-behind children, respectively, controlling for children’s ages and reasoning abilities. Among non-left-behind children, boys scored higher than girls, *F*(1,108) = 6.11, *p* = 0.015, $\eta_{p}^{2}$ = 0.054, while among left-behind children, girls scored higher than boys, *F*(1,97) = 4.60, *p* = 0.035, $\eta_{p}^{2}$ = 0.045. Another two ANCOVAs were used to examine the group differences in boys and girls, respectively, controlling for children’s ages and reasoning ability. Left-behind girls performed better than non-left-behind girls, *F*(1,98) = 4.30, *p* = 0.041, $\eta_{p}^{2}$ = 0.042, while left-behind boys scored lower than non-left-behind boys, *F*(1,107) = 6.20, *p* = 0.014, $\eta_{p}^{2}$ = 0.055. The interaction between gender and group is illustrated in **Figure 1**.

For the faux pas understanding, the main effect of gender, *F*(1,207) = 0.03, *p* = 0.869, $\eta_{p}^{2}$ < 0.001, and the gender × group interaction, *F*(1,207) = 0.96, *p* = 0.329, $\eta_{p}^{2}$ = 0.005, were not significant, but the main effect of group was significant, *F*(1,207) = 4.24, *p* = 0.041, $\eta_{p}^{2}$ = 0.020. Non-left-behind children scored higher than left-behind children.

**FIGURE 2:**
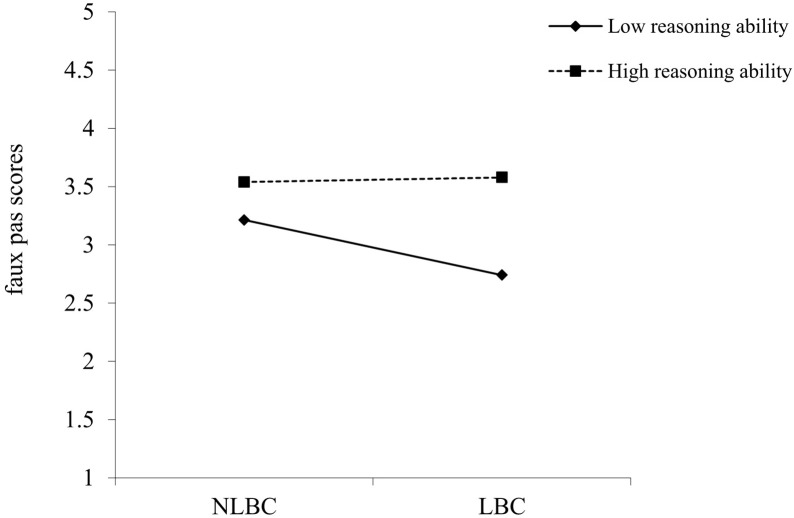
The two-way interaction of group and reasoning ability on children’s faux pas understanding. LBC represents left-behind children and NLBC represents non-left-behind children.

Finally, several hierarchical regressions on each theory of mind task score were conducted individually to examine the potential protective effect of general reasoning ability. All variables were standardized before being entered into the regression. For the regression on faux pas understanding (**Table 3**), standardized age, gender, group, and general reasoning ability were entered into step 1, *R*^2^ = 6.7%, *F*(4,208) = 3.74, *p* = 0.006, and the interaction between group and general reasoning ability was entered in the second step, Δ*R*^2^ = 2.7%, Δ*F*(1,207) = 6.16, *p* = 0.014. Then, procedures by Aiken and West (1991) and Dawson (2014) were adopted to plot the interaction effects and to test for significant differences between the slopes. **Figure 2** plots the interactive effect between children’s group and general reasoning ability on their faux pas understanding. For children with high levels of general reasoning ability, left-behind children and non-left-behind children performed similarly on the faux pas task (*gradient of simple slope* = 0.04, *t* = 0.26, *p* = 0.793), while for children with low levels of general reasoning ability, left-behind children scored lower than non-left-behind children (*gradient of simple slope* = −0.473, *t* = 3.19, *p* = 0.002). These results indicated that general reasoning ability conferred stability in children’s faux pas understanding despite the increased risk (Luthar et al., 2000).

**Table 3:** Hierarchical regression on children’s faux pas understanding.

	*B*	*SE*	β	*t*	*p*
**Step 1**					
Age	0.25	0.10	0.17	2.45	0.015
Gender	−0.01	0.10	−0.01	−0.10	0.919
Group	−0.22	0.11	−0.15	−2.12	0.035
Reasoning ability	0.16	0.11	0.11	1.56	0.121
**Step 2**					
Age	0.27	0.10	0.17	2.59	0.010
Gender	−0.02	0.10	−0.01	−0.19	0.847
Group	−0.22	0.10	−0.14	−2.10	0.037
Reasoning ability	0.16	0.10	0.11	1.58	0.117
Group × reasoning ability	0.256	0.11	0.16	2.48	0.014

*Gender and group were dummy variables, and 1 represents boys, while 0 represents girls; 1 represents left-behind children, while 0 represents non-left-behind children in this study.*

## Discussion

This study was the first to investigate the effects of the left-behind experience on school-aged children’s theory of mind development. It found that left-behind children were disadvantaged on both the faux pas task and Strange Stories task vis-à-vis those from non-migrant families in the same rural communities, even controlling for their ages and general reasoning ability. Interestingly, we also found that on the second-order false belief task, left-behind boys scored lower than non-left-behind boys, while left-behind girls scored higher than non-left-behind girls. These results indicated that the left-behind experience had a negative effect on children’s theory of mind development and especially played a negative role in boys’ second-order false belief understanding. Finally, we found that children’s general reasoning ability moderated the relationship between parental migrant status and children’s faux pas understanding: for children with low levels of general reasoning ability, left-behind children’s faux pas understanding was lower than that of non-left-behind children, but for children with high levels of general reasoning ability, left-behind children’s faux pas understanding was similar to that of their counterparts, indicating that general reasoning ability might buffer the negative effect of being left behind on children’s faux pas understanding.

To date, few empirical studies have examined the theory of mind development of left-behind children in comparison with that of non-left-behind children in rural China. Previous studies have documented that the frequency of parent–child communication is lower for left-behind rural children than for non-left-behind rural children (Zhang and He, 2013); most migrant parents give their left-behind child a call every half month, lasting only a few minutes (Li, 2002). Moreover, migrating parents communicate simply with their left-behind children, often only discussing children’s learning situations rather than their emotional states or peer interaction. According to social cognitive theory, social experience with their parents is important for children’s theory of mind development. While communicating with their parents, children may be exposed to many perspectives and may realize that different people can have different views of the same situation (Carpendale and Lewis, 2004; Peterson and Slaughter, 2006; Nelson, 2007; Pavarini et al., 2013; Hao and Su, 2014; Bianco et al., 2016), which can facilitate children’s theory of mind development. Thus, the limited social experience with their parents may give left-behind children less exposure to different perspectives than their non-left-behind counterparts, which led to left-behind children’s poorer performance on the faux pas task and Strange Stories task.

Interestingly, this study found that girls could capitalize on the adversity of being left behind and thrive in second-order false belief understanding. According to Maccoby (1966), gender differences in children’s peer interactions cause subsequent differences in social cognition. In middle childhood, gender-segregated peer play is typical, with more conversations and emotional closeness in girls’ peer interactions than in boys’ play (Banerjee et al., 2006; Pasterski et al., 2010). Due to limited parental supervision, left-behind girls spend more time communicating with their peers than their counterparts (Sun et al., 2010), which may provide more opportunities for them to practice their theory of mind. More research is needed to explore the interaction effects between family socialization and gender on child development and the underlying mechanism (Wen and Lin, 2012).

Additionally, this study indicated that general reasoning ability buffered the negative effect of being left behind on children’s theory of mind development. On the one hand, left-behind children with advantages in general reasoning could appreciate and accept parental migration for work (Sison and Arellano-Carandang, 2007), and thus they may initiatively seek the guidance and support needed for their development. On the other hand, teachers would provide more care and conversations for the students with good general reasoning ability, and this is especially true for those who were left-behind by their parents (Liu et al., 2015). Thus, despite limited the communication with parents, such characteristics of left-behind children with high levels of general reasoning ability would help them develop their theory of mind though another ways. The findings that general reasoning ability played a protective role in left-behind children’s faux pas understanding are encouraging: it is likely to be easier for school educators to improve general reasoning ability via school intervention to facilitate left-behind children’s theory of mind development than to improve parent–child communications, which is hard to influence migrant parents in rural China. Overall, these results provide empirical support for the resilience theory for child development. According to this theory, adversity does not always have negative effects on child development, and children from high-risk populations also have opportunities for normal development; some children may even outperform those without a disadvantaged environment when they also have protective factors (Luthar et al., 2000; Lerner et al., 2009).

It should be noted that in our study the protective roles of gender and general reasoning ability in left-behind children’s theory of mind development were found in different theory of mind tasks. This may be due to the complex nature of theory of mind (Brizio et al., 2015). Researchers have increasingly emphasized the necessity of distinguishing cognitive theory of mind from affective theory of mind (Abu-Akel and Shamay-Tsoory, 2011; O’Brien et al., 2011; Wang and Su, 2013; Hao and Liu, 2016). Cognitive theory of mind involves an understanding of beliefs and intentions, whereas affective theory of mind refers to an understanding of emotions and feelings. In our study, the second-order false-belief task tested the cognitive component of theory of mind, while faux pas task measured the affective component of theory of mind.

The current study contains some limitations. First and foremost, the cross-sectional design prevents us from drawing any causal conclusion regarding the relationship between parental migrant status and children’ theory of mind and from describing left-behind children’s theory of mind developmental trajectory. Second, the sample was from rural communities in Hubei Province of central China. The conclusions should be generalized to other populations with caution due to the nonrandomized sampling method adopted in this study. Moreover, this study focused only on the protective role of general reasoning ability in left-behind children’s theory of mind. It is necessary for future research to examine the potential protective effects of other factors (e.g., peer companionship or communications, parent–child relationship) on children’s theory of mind. Finally, this study focused on the effect of being left behind on theory of mind in middle childhood; therefore, it is unclear whether and how the left-behind experience affects adolescents’ theory of mind development. Due to frequent communication with peers and decreased parent–child communication during adolescence, left-behind children’s theory of mind development might catch up with their counterparts. Given the importance theory of mind plays in individuals’ academic achievement and social life during adolescence (Brizio et al., 2015), it is necessary to investigate the development and mechanisms of theory of mind in left-behind adolescents.

This study contributes to the literature of school-aged children’s theory of mind by comparing left-behind children of migrant parents with non-left-behind children in rural areas of mainland China. On the one hand, this study found that parental migration for work might not only dampen children’s performance on the Strange Stories task and faux pas understanding but also negatively predict boys’ second-order false belief understanding; this finding provides empirical support for the social cognitive theory concerning the effects of parental communication absence on children’s theory of mind. On the other hand, the study found that children’s general reasoning ability might buffer the negative effect of being left behind on children’s theory of mind, indicating that risk factors do not always have negative effects on children’s development; this finding provides empirical support for the resilience model of child development.

## Author Contributions

YL proposed the conception, designed the work, and wrote the manuscript. XY, JL, EK, HT, and HH performed the data acquisition, analysis, and interpretation. All the authors revised the work for important intellectual content, approved the final version to be published, and agreed to be accountable for all aspects of the work.

## Conflict of Interest Statement

The authors declare that the research was conducted in the absence of any commercial or financial relationships that could be construed as a potential conflict of interest.

## Footnotes

1 www.jeremydawson.com/slopes.htm
